# Il Silenzio: The First Renaissance Oil Painting on Canvas from the Uffizi Museum Restored with a Safe, Green Antimicrobial Emulsion Based on *Citrus aurantium* var. *amara* Hydrolate and *Cinnamomum zeylanicum* Essential Oil

**DOI:** 10.3390/jof8020140

**Published:** 2022-01-29

**Authors:** Debora Minotti, Lara Vergari, Maria Rita Proto, Lorenzo Barbanti, Stefania Garzoli, Francesca Bugli, Maurizio Sanguinetti, Luigia Sabatini, Alice Peduzzi, Roberto Rosato, Maria Grazia Bellardi, Paola Mattarelli, Daphne De Luca, Maura Di Vito

**Affiliations:** 1 Department of Pure and Applied Sciences (DiSPeA), University of Urbino Carlo Bo, Piazza della Repubblica, 13, 61029 Urbino, Italy; debora.minotti@uniurb.it (D.M.); laravergari@gmail.com (L.V.); daphne.deluca@uniurb.it (D.D.L.); 2 Department of Agricultural and Food Sciences, University of Bologna, Viale G. Fanin 42, 40127 Bologna, Italy; mariarita.proto2@unibo.it (M.R.P.); lorenzo.barbanti@unibo.it (L.B.); mariagrazia.bellardi@unibo.it (M.G.B.); paola.mattarelli@unibo.it (P.M.); 3 Dipartimento di Chimica e Tecnologie del Farmaco, Università di Roma Sapienza, Piazzale Aldo Moro 5, 00100 Rome, Italy; stefania.garzoli@uniroma1.it; 4 Dipartimento di Scienze Biotecnologiche di Base, Cliniche Intensivologiche e Perioperatorie, Università Cattolica del Sacro Cuore, Largo A. Gemelli 8, 00168 Rome, Italy; francesca.bugli@unicatt.it (F.B.); maurizio.sanguinetti@unicatt.it (M.S.); roberto.rosato01@icatt.it (R.R.); 5 Dipartimento di Scienze di Laboratorio e Infettivologiche, Fondazione Policlinico Universitario A. Gemelli IRCCS, Largo A. Gemelli 8, 00168 Rome, Italy; 6 Dipartimento di Scienze Biomolecolari, Sezione di Farmacologia e Igiene, Università Degli Studi di Urbino Carlo Bo, 61029 Urbino, Italy; luigia.sabatini@uniurb.it; 7 Dipartimento di Biologia ambientale, Sapienza Università di Roma, Piazzale Aldo Moro 5, 00185 Roma, Italy; alice.peduzzi@gmail.com

**Keywords:** *Citrus aurantium* var. *amara*, *Cinnamomun zeylanicum* bark, paint artwork, biodeteriogens, safe restoration

## Abstract

Preserving artworks from the attacks of biodeteriogens is a primary duty of humanity. Nowadays, restorers use chemicals potentially dangerous for both artworks and human health. The purpose of this work was to find a green and safe formulation based on natural substances with fungicidal activity to restore ancient oil paintings, particularly “Il Silenzio” (by Jacopo Zucchi) preserved at the Uffizi Museum in Florence, Italy. The study was divided into two phases. First phase (in vitro study): three essential oils (EOs) and four hydrolates (Hys) were analysed by GC-mass spectrometry and in vitro tested against six ATCC strains of molds. An emulsion based on the more active natural compounds was tested on aged and unaged canvases samples to evaluate both their fungicidal activity and the impact on chemical-physical parameters. Finally, an in vivo toxicity test performed on the *Galleria mellonella* model assessed the safety for health. Second phase (in situ application): the emulsion was sprayed on the back of the painting and left to act for 24 h. Biodeteriogens present on the “Il Silenzio” painting were microbiologically identified before and after the treatment. The emulsion formulated with *C. zeylanicum* EO and *C. aurantium* var. *amara* Hy showed the best antifungal activity both in vitro and in situ without altering the chemical-physical characteristics of paintings. Furthermore, no in vivo toxicity was shown. For the first time, a green antimicrobial emulsion based on Hy and EO, safe for operators, was used to decontaminate an artwork colonised by fungi before the restoration practices.

## 1. Introduction

All microbial agents that affect artwork will cause irreversible damage. Therefore, it is essential to monitor these contaminations in order to avoid damage such as increased fragility, disconnection, change of colour, or complete destruction of the canvas painting [1]. The biodeterioration control of cultural heritage materials involves measures to stop the degradation caused by both microorganisms and organisms and, whenever possible, to delay its reappearance. All artefacts, with time, are subject to attack by biodeteriogens. Paper, vegetable/animal fibres, wood, and painted artworks are mainly attacked by fungi due to their cellulose content, while stone colonisation follows a precise pattern: fungi prior to algae, mosses, and lichens and common organisms (from cryptogams to higher plants). Furthermore, fungi represent both a serious danger to artefacts and a risk to human health. In addition to the prevention practices, those of disinfection are carried out before any other manipulation to avoid the spreading of spores, which are potentially dangerous for both artworks and human health. To prevent or treat colonisation by fungi, restorers use chemicals such as benzalkonium chloride (alkyl dimentylbenzylammonium chloride), parachloromethacresol, 3-methyl-4-chlorophenol, etc. The most used products in the restoration of paintings are Preventol^®^ RI80/RI50, Biotin R, and Biotin T. Preventol^®^ RI80/RI50 is a concentrated formulation of quaternary ammonium salts with a broad spectrum of activity against fungi, bacteria, and algae, and is generally diluted from 2% to 5% in water or ethanol. Biotin R is a formulation characterised by iodiopropynylbutylcarbamate (IPCB) and n-octyl-isothiazolinone (OIT), dissolved in 2(2-butoxy-ethoxy) used at 3% in ligroin, petroleum essence or white spirit, while Biotin T is composed of OIT and a quaternary ammonium salt used at 3% in demineralised water, ethyl alcohol, or butyl acetate [2,3,4,5]. Chemicals generally used in these formulations can cause serious problems to ecosystem balance and human health such as mutagenicity (ability to induce genetic mutations), carcinogenicity (ability to induce cancer), teratogenicity (ability to induce abnormalities in embryonic development), and embryotoxicity (ability to induce toxic effects to the embryo), but also sensibilisation or allergic reactions against specific chemicals or additives [5,6,7]. In order to identify new cytocidal products with low environmental impact and high human safety, different research groups are turning their attention to the potential of both essential oils (EOs) and hydrolates (Hys) that are mixtures of natural substances obtained through the distillation of vegetable parts of aromatic plants [8,9,10,11]. The former are extremely concentrated hydrophobic natural products with an important antimicrobial activity, and that cannot be used pure because they are potentially toxic. Several research groups have tested the antimicrobial activity of EOs especially in the restoration of marble artworks, while studies of their applications on other types of cultural heritage such as paper, textile, or painting artworks are limited [12,13,14,15,16,17,18,19,20,21,22]. Whereas, the latter (very cheap products of distillation) are extremely diluted hydrophilic mixtures with lower microbicidal activity in exchange for a higher versatility and safety. Hys have only recently been discovered in cultural heritage environment because their characteristics make them less aggressive than EOs and applicable in contexts where EOs cannot be applied, such as the restoration of paper works [23].

The purpose of this work was to identify in vitro a mixture based on hydrolats and/or essential oils to be used in a spray formulation for the in situ restoration of “Il Silenzio” (by Jacopo Zucchi), an oil on canvas painting preserved at the Uffizi Museum in Florence.

## 2. Materials and Methods

### 2.1. Fungal Strains

The fungal strains studied belonged to biodeteriogenic species frequently isolated on pictorial artworks. The fungal strains investigated were *Aspergillus niger* (ATCC9642), *Aureobasidium pullulans* (ATCC 15233), *Chaetomium globosum* (ATCC 6205), *Cladosporium cladosporioides* (ATCC 16022), *Alternaria alternata*, and *Penicillium citrinum* from the fungal collection in the Urbino University Carlo Bo Biomolecular Sciences Department.

### 2.2. Essential Oils (EOs) and Hydrolates (Hys)

Three EOs and four Hys were tested. *Cinnamomum zeylanicum* EOs were supplied by Pranarom International (Ghislenghien, Belgium). Whereas the EOs of *Monarda didyma* and *Monarda fistulosa* together with the Hys of *M. didyma*, *M. fistulosa*, and *Monarda citriodora* were supplied by the Dept. of Agricultural and Food Sciences, University of Bologna, Italy. The *Citrus aurantium* var. *amara* Hy was supplied by Erboristeria Magentina s.r.l. (Turin, Italy).

### 2.3. Canvas Samples

To simulate the execution techniques of paintings on ancient canvas, two types of canvas samples were created as described in Table 1. All samples were used for in situ experiments; specifically, the first type of samples was used for chemical-physical analyses (Section 2.8), while the second one for microbiological analysis (Section 2.7).

**Figure 1:**
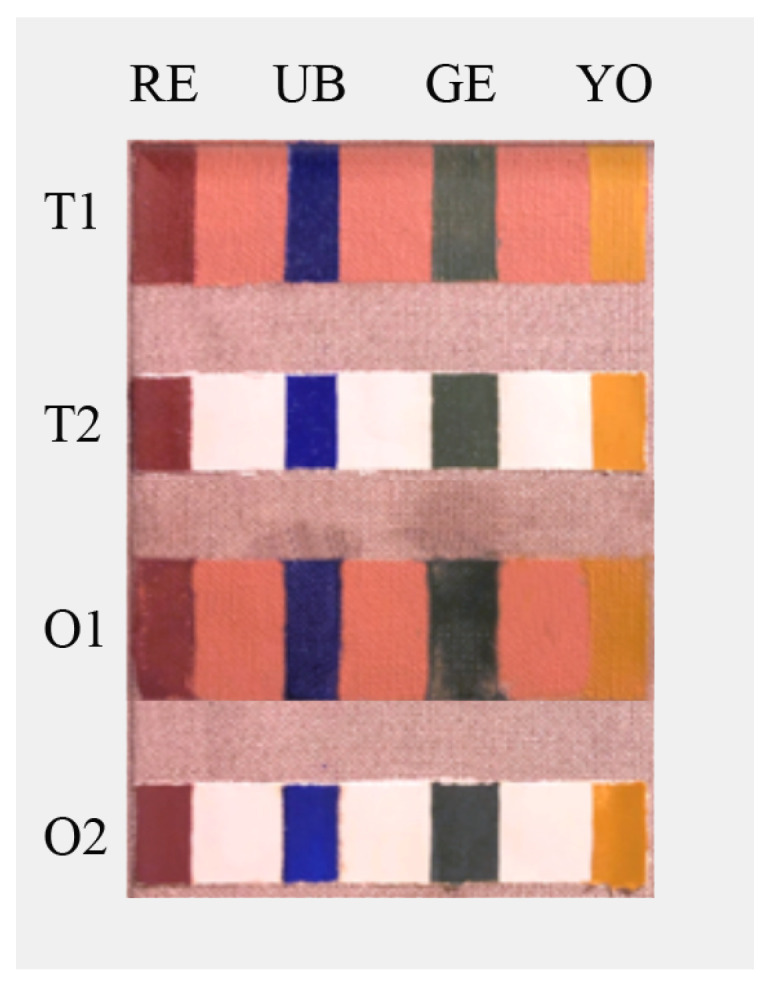
Big Canvas Samples used for in situ experiment to evaluate chemical-physical changes before and after treatment. T1, T2, O1, and O2 formulated as described in Table 1. RE: red earth, UB: ultramarine blue, GE: green earth, YO: yellow ochre.

Figure 1 show the first type of samples that were two big canvas samples (BCS) simulating ancient painting techniques (in the text named T1—T2—O1—O2) created as described in Table 1. The specimen support was a rough canvas with linen flap and treated with 2 layers of rabbit glue 1:12. The canvas was then stretched on a custom-made wooden frame (26 cm high × 18 cm wide × 2.5 cm thick) using a continuous fastening system. The second type of samples comprised 51 small canvas squares (SCS) of 2 cm × 2 cm, performed with ancient techniques (dressing and preparation of chalk and glue) and used for the in vitro microbiological test.

### 2.4. Gas Chromatography and Mass Spectroscopy Analysis (GC-MS)

The chemical analysis of *C. zeylanicum* EO was carried out with the use of a Gas Chromatograph coupled with a single quadrupole Mass Spectrometer (model Clarus 500-Perkin Elmer, Harfsen, The Netherlands). The analysis was repeated in triplicate. The chemical compositions of other EOs and Hys obtained with the same procedure have already been published by our group [23,24,25]. The GC was equipped with a Varian Factor Four VF-1 capillary column. Helium was used as a gas carrier at a flow of 1 mL/min and the operative condition of an injector temperature 280 °C; oven GC temperature ramp: 60 °C for 5 min and ramped to 220 °C at a rate of 5 °C/min for 20 min. One mL of EO was diluted in 1 mL of methanol and 1 mL of this solution was injected. The Ionisation energy of MS was 70 eV and the scan range was 40–450 *m*/*z*. The ion source and the connection parts temperature were 200 °C and 220 °C respectively. Identification of separated compounds was performed based on Linear Retention Indices (LRIs) calculated with reference to the series of n-alkanes (C8–C30 aliphatic hydrocarbons, Ultrascientific s.r.l. Bologna, Italy) injected under the same experimental conditions and by the comparison of mass spectra with those of authentic standards from Wiley and Nist libraries. The quantification of the identified compounds was obtained by GC-FID analysis using the same column at the same conditions reported above. Relative percentages were obtained by peak area normalisation without the use of an internal standard or correction factors.

### 2.5. Broth Microdilution Susceptibility Test

According to the International Guidelines EUCAST [26], micro-broth dilution test was used to identify fungal sensitivity to both EOs and Hys. The test was performed on 96-well plates in a total volume of 100 μL by using RPMI-1640 (Sigma-Aldrich, St. Louis, MO, USA) broth as culture medium. Concentrations between 2% *v*/*v* to 0.06% *v*/*v* and 50% *v*/*v* to 1.6% *v*/*v* were tested respectively for EOs and Hys against a fungal suspension of 2.5 × 10^5^ CFU/mL. Plates were incubated for 7 days at 30 °C and the minimum inhibitory concentration (MIC) was determined. MIC is defined as the lowest concentration of the substance at which fungi showed no visible growth, as compared with control. To evaluate the minimum fungicidal concentrations (MFC), sub-cultures were made by seeding 20 μL of each well content on Potato Dextrose Agar (PDA) (Oxoid, Basingstoke, UK). MFC was defined as the lowest concentration of the substance corresponding to the death of 99.9% or more of the initial inoculum. Each test was performed in triplicate, and both negative and positive controls were included.

### 2.6. Spray Formulation

An emulsion characterised by the Hy and the OE with greater in vitro anti-fungal efficacy was formulated. To identify the best spray formulation to be used for in situ experiments, scalar dilutions of the *C. zeylanicum* EO (from 1% *v*/*v* to 0.07% *v*/*v*) in *C. aurantium* var. *amara* Hy were performed. The dilutions were vortexed for 1 min and placed in a quiet position for 3 h; at the end, the first emulsion showing the lowest concentration of the EO completely dissolved in the Hy, without any phase separation, was chosen.

### 2.7. In Situ Microbiological Test

The SCS were used to evaluate the in situ fungicidal activity of the spray formulation performed as described in paragraph 2.6. Before starting, all canvases were sterilised by UV light for 80 min (40 min per side). Then, 50 mL of a fungal suspension of 2.5 × 10^5^ CFU/mL (equal amount of *A. niger*, *A. pullulans, C. globosum*, *C. cladosporioides*, *A. alternata*, and *P. citrinum*) was inoculated on the back of the canvases. After drying, from 1 to 5 sprays (capacity of 55 μL/spray, i.e., 14 mL/cm^2^ of each SCS) of the formulation were applied on the back ok SCS for 3, 5, or 24 h. The treated side of canvas was placed on PDA (Oxoid, Basingstoke, UK) and incubated for 7 days at 30 °C. At the end of the incubation period, treatment effectiveness was evaluated. Untreated canvas (positive control) and uninoculated canvas (negative control) were included in tests. Each treatment was repeated in triplicate.

### 2.8. Chemical-Physical Analysis

To assess if the spray mixture application (view Section 2.6) could cause changes in the chemical-physical or structural state of the canvas, preparatory layers, and/or paint film, specimens with and without aging were subjected to chemical and physical analysis (view the following paragraphs) before and after the anti-microbial treatment.

#### 2.8.1. Ageing Conditions

To evaluate the effectiveness of the treatment over time, BCS were aged for 3 weeks using “Solar box 3000e RH Xenon” (equipped with a xenon lamp, outdoor filter (280 nm) and radiation power of 550 W/m^2^). Ageing was carried out using hot/humid conditioning cycles at 30 °C and relative humidity between 55% and 99% for 72 h, interspersed with atmospheric humidity cycles of 55% for a duration of 96 days. The cycle was repeated continuously throughout the overall ageing duration of the specimens (3 weeks).

#### 2.8.2. pH Measurements

To evaluate the impact of the treatment on the pH of canvases, tests according to the Cremonesi (2012) method [2,27] were performed. Briefly, 3 discs (3 mm of diameter) of 2% (*w*/*v*) agar (AGAR AGAR—ANTARES, Bologna, Italy) were placed on the surface of each specimen for 3 min. Using the “ISFET Mini-Lab^®^” pH was later evaluated by placing the disc in direct contact in with the sensor. The difference between the values obtained before and after natural treatment on both aged and unaged canvas was then calculated; its statistical strength was assessed by submitting the data before and after treatment for each colour and painting technique to a t-test which was run using the CoStat 6.3 statistical software (Cohort, Monterey, CA, USA).

#### 2.8.3. Colorimetric Measurements

Colorimetric measurements were carried out with the “Konica Minolta CM-2600d” (with diffuse illumination, 8-degree display with simultaneous measurement of specular component included, SCI, and specular component excluded, SCE, with γ rays, λ 360–740 nm). Colorimetric data were reported as a function of standard CIE D_65_ illumination and 10° (CIE 1964) supplementary standard observer excluding the specular component of radiation. The colorimetric coordinates L*, a*, b* were calculated as a function of the CIEL*a*b 1976 colour space [28]. Differences in colour (ΔE), chroma (ΔC), and brightness (ΔL), together with the mean (M) and standard deviation (SD), were calculated starting from L*, a*, b* values according to the 2000 CIE guidelines [29]. Measurements were taken in triplicate. Each Δ value was interpreted as follows: 0–1 (colour difference not detectable by the human eye), 1–3 (small colour difference), 3–6 (perceptible difference), or >6 (large difference). Only values greater than 3 were assessed as significant because values ≤3 are not detectable or are considered irrelevant [30].

#### 2.8.4. Spray Treatment

The canvas samples, carefully removed from the support, were placed in a glass container, with the paint layer facing downwards, separated from the glass bottom by a Melinex^®^ sheet. A total of 50 sprays (14 mL/cm^2^) of freshly prepared mixture (Section 2.5) were used for the treatment. Twenty-five sprays were applied to the back of the canvas samples and 25 on acid-free absorbent paper sheets placed in contact with the back of the canvas. Finally, five weights were placed on the top of the absorbent paper in order to avoid the movement of the textile fibres and to better keep both surfaces in contact with each other. Lastly, the glass container was sealed by a special lid, from which evaporation of the volatile components was prevented, and incubated at 30 °C for 5 h. The treatment was performed in triplicate.

### 2.9. Toxicity Study

The possible toxicity of the emulsion was evaluated in an in vivo model of *Galleria mellonella* [31]. Larvae with colour changes in the body and larvae outside the body weight of 0.2–0.3 g were excluded. Ten (10) μL of the emulsion of *C. zeylanicum* EO (bark) (0.03% *v*/*v*) in *C. aurantium* var. *amara* hydrosol were injected into the haemocoel through the last left pro-leg of ten larvae using a 0.5 mL syringe. The puncture area was decontaminated with 70% ethanol prior to administration. The larvae were then placed in an aerobic incubator at 33 °C and observed every 8 h for 3 days.

### 2.10. Treatment of the Museum Painting

**Figure 2:**
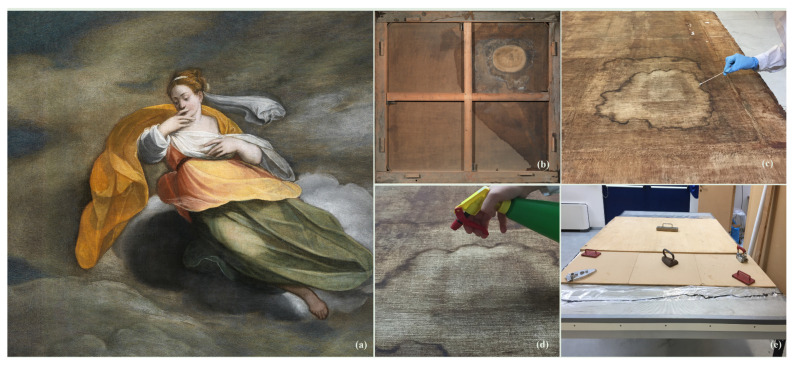
Restoration phases of the “Il Silenzio” painting. (**a**) Canvas after restoration. (**b**) Microbiological attack on the back of the canvas before restoration. (**c**) Collection of microbiological samples carried out on the back of the canvas with cotton swabs. (**d**) Spray application of the emulsion based on *C. aurantium* var. *amara* hydrolate and Cinnamomum zeylanicum essential oil. (**e**) Artwork on the low-pressure table during treatment.

The painting named “Il Silenzio” by Jacopo Zucchi, pupil of Giorgio Vasari (Figure 2a, year: 1572, dimensions: 146 cm × 163 cm, oil on canvas) exhibited on the ceiling of the “Room of the Geographic Maps” of the Uffizi Museum (Florence, Italy) was subjected to the cytocidal treatment using the spray formulation previously selected by in vitro and in situ tests carried out on ancient canvas models.

#### 2.10.1. Sampling and Microbial Analysis of the Artwork

To identify the microbial strains responsible for biodeterioration, fifteen samples were collected before the spray treatment. Particularly, five cotton swabs (Boettger, Paul Boettger GmbH & Co. KG, Bodenmais, Germany) and five fungi-tapes (Scientific Device, Glenview, IL, USA) were collected on the back of the artwork (Figure 2b), while only five cotton swabs were collected on the front. All samples were picked up in correspondence with the area potentially characterised by biodeteriogens’ attack (Figure 2c) and were sent to the microbiology laboratory. Afterwards, samples were seeded on the following nutrient agar: Muller Hilton agar, Malt Extract agar, and PDA (all by Oxoid, Basingstoke, UK) and incubated for 7 days at 30 °C. At the end of the incubation time, the recognition of the fungal strains was carried out using matrix-assisted laser desorption ionisation time-of-flight mass spectrometry (MALDI-TOF MS) Bruker Daltonics (Bruker, Bremen, Germany).

#### 2.10.2. Spray Cytocidal Treatment

Before treatment, the artwork was released from the frame and the canvas was removed to uniformly administer the treatment. The artwork was placed on a low-pressure table (Series: NSD 1101 by CTS srl Altavilla Vicentina, Vicenza, Italy) on which a sheet of Melinex^®^ (Monosiliconated polyester film thickness 23-micron, weight 33 g/m^2^ Cts srl Altavilla Vicentina, Vicenza, Italy) had previously been spread. Three-hundred mL of mixture (about 14 mL/cm^2^), consisting of 299.1 mL (99.7% *v*/*v*) of *C. aurantium* var. *amara* Hy and 0.9 mL (0.3% *v*/*v*) of *C. zeylanicum* EO, were freshly prepared. The mixture was applied by spray (Figure 2d) after mixing it to obtain a uniform emulsion. A total of 96 sprays were delivered on the artwork. Particularly, 48 sprays were applied on the back of the canvas and the other 48 on a sheet of blotting paper (acid-free absorbent paper 300 g/m^2^ Vangerow srl, Bolzano, Italy), which was placed in direct contact with the back of the painting. Each spray had a capacity of 3 mL and was delivered at about 20 cm from the surface. To avoid the dispersion of the active compounds of both EO and Hy, the Melinex^®^ was folded, enclosing within it both the artwork and the blotting paper by forming a container bag sealed with insulating tape. The treatment was performed for 24 h, 7 of which on the low-pressure table by setting only the temperature parameter (30 °C), and the remaining time at RT. Furthermore, a sheet of plywood was placed on the canvas and stopped with six weights (from 150 to 500 g) to avoid movements of pictorial films of canvas (Figure 2e). The next day the Melinex^®^ container was opened, the blotting paper was removed, and five swab samples were taken on the back of the canvas. Afterwards, the remaining low degree of humidity was removed by operating the low-pressure table (40 hPa and 30 °C for 30 min) and the subsequent stages of restoration were initiated.

## 3. Results

### 3.1. GC/MS Analysis

Table 2 listed the twenty-four compounds of *C. zeylanicum* EO identified by GC/MS analysis. The chemical composition of the *C. zeylanicum* EO was characterised by cinnamaldehyde (66.0%) as a major compound followed by β-caryophyllene (5.8%), (*E*)-cinnamyl acetate (5.5%), linalool (4.9%), 1,8-cineole (4.4%), and eugenol (4.1%). Several minor compounds with a percentage value from 0.1% to 0.4% were also detected. The chemical compositions of other EOs and Hys have already been analysed by our group [23,24,25].

### 3.2. Broth Microdilution Susceptibility Test

Table 3 shows the results of the in vitro broth microdilution susceptibility tests.

Among the OEs tested, the OE of *C. zeylanicum* was the most effective in terms of both MIC and MFC values (0.008% *v*/*v* and 0.125% *v*/*v* respectively). Similarly, the Hy of *C. aurantium* var. *amara* was the most effective of Hys, showing MIC and MFC values equal to 6.25% *v*/*v* and 3.12 *v*/*v*.

### 3.3. Microbiological Tests on Canvas Samples

SCS were treated as described in paragraph 2.3 by using a spray emulsion composed of *C. zeylanicum* EO (0.03% *v*/*v*) dispersed in *C. aurantium* var. *amara* Hy. The spray solution was selected and applied as specified in Section 2.6 and Section 2.7. At the end of the incubation periods, all canvas samples, including those treated with the lowest concentration and shorter time exposure (one sprays for 3 h), showed the total absence of fungal growth. The data indicated the efficacy of the treatment even at the lowest concentrations.

### 3.4. Artificial Ageing

After artificial ageing, BCS samples showed visible colour changes in cohesion and adhesion subsequently confirmed by the colorimetric analysis (see Section 3.6). The T1 was more sensitive than T2 to humidity cycles due to the different rheological characteristics. Whereas, no significant variations were observed on both O1 and O2 samples. The support had a more yellowish colour due to the polymerisation and aging of the textile fibres.

### 3.5. pH

Table 4 reports data carried out on pH analyses. Variations of pH after treatment were less than one unit of pH and therefore acceptable for both aged and unaged samples. In the unaged samples, the greatest variations were related to the red and ochre colours of the tempera on priming. Whereas, the treatment on treated, aged samples resulted in significant and beneficial alkalinisation of the BCS surface except for the green earth colour.

### 3.6. Colorimetric Measurements

Table 5 shows the average values of the L*a*b values obtained before and after the treatment on unaged and aged samples. The ΔE data showed that unaged samples did not undergo significant colour change after treatment (ΔE ≤ 3), while among the aged samples those made of tempera exhibited significant colour variation after treatment. Specifically, the T1 samples showed red, blue, and green variations, while the T2 samples showed only a blue variation. The O1 and O2 samples were unaltered. Therefore, the data indicated reasonable safety of use for oil paintings.

### 3.7. Toxicity Study

*Galleria mellonella* larvae treated with 10 mL of the emulsion were monitored every 8 h for three days. On the third day, all larvae were perfectly viable, and none showed signs of toxicity (colour change). In the selected in vivo model, data showed no toxicity of the treatment based on the emulsion studied.

### 3.8. Treatment of “Il Silenzio” Painting

#### 3.8.1. Microbiological Test

Microbiological tests performed on samples picked up in correspondence of both the front and the back of the area potentially characterised by biodeteriogens’ attack showed the presence of *Aspergillus versicolor* and *Penicillium chrysogenum* only on the back of the painting.

#### 3.8.2. Cytocidal Treatment

The treatment was performed by the restorer without any methodological problem or operator’s health problems. These data supported the safety data obtained from the in vivo model of *Galleria mellonella* (see Section 3.7). At the end of the treatment, no visual changes of the painting were detected by the operator. Microbiological test carried out on samples picked up after removal of the blotting papers showed the absence of any microbial growth.

## 4. Discussion and Conclusions

In recent years, more and more research has been focused on the use of EOs in the treatment of several types of artworks. In fact, we have to consider that all materials employed and exposed in historical and cultural institutions (i.e., museums, archives, libraries) are excellent substrates for fungal growth in the presence of favourable micro-climate conditions such as poor ventilation, humidity, pH, temperature, etc. Recently, both *Origanum vulgare* and *Thymus vulgaris* EOs have been studied to counteract the colonisation of biodeteriogens and the infestation of insects on wood and algae, mosses, and lichens on stone artworks [12,13,14]. Other studies have directly investigated the activity of single components of EOs by identifying which of them (thymol and eugenol) were suited for formulations applicable in the restoration of wall paintings [15]. Very few studies have involved the application of formulations based on EOs directly on artworks. Most of the scientific articles are in vitro or ex situ studies. Stupar et al. [12] tested the efficacy of *O. vulgare* EO on biodeteriogenic fungal strains directly isolated from the Serbian monastery Holy Virgin Church [12]. Whereas Borrego et al. [16] showed the effectiveness of *Syzygium aromaticum*, *Allium sativum*, and *O. vulgare* EOs against biodeteriogens isolated from the air inside the repositories and documents of the National Archive of the Republic of Cuba and the Historical Archive of the Museum of La Plata, Argentina [16].

In vitro studies showed cytocidal activity of other EOs such as those of *Cinnamomum camphora*, *Helichrysum Italicum*, *Lavandula angustifolia*, and *Cymbopogon winterianus* against dangerous fungal strains isolated from artworks [13,17]. There are very few studies concerning the use of *C. zeylanicum* EO in cultural heritage. Matusiak et al. [18] reported the use of these EOs for the disinfection of textiles heritage. The study showed the bactericidal and fungicidal activity of vapours of *C. zeylanicum* EO in disinfecting textiles without altering their chemical-physical characteristics. In a recent work, Campanella et al. [19] showed the potential use of *C. zeylanicum* EO when encapsulated in psyllium-alginate beads for applications on paper works [19]. However, the Hy of *C. aurantium* var. *amara* (flowers), when used alone or incorporated in gellan gel, has already shown a fungicidal activity against fungal strains harmful to the paper heritage. The data of this study were consistent with those of the literature. Both *C. aurantium* var. *amara* hydrolate and *C. zeylanicum* essential oil showed the best cytocidal activity against potentially pathogenic fungal strains for pictorial artworks.

For the first time, the cytocidal activity of an emulsion spray based on one EO and one Hy (named *Zeylantium green* emulsion) was studied in vitro on canvas samples, in vivo on animal model of *Galleria mellonella*, and in situ directly on an important artwork exhibited to the public in one of the most prestigious museums in the world.

The in vitro experiments conducted on canvas samples, aged and unaged, indicated that the treatment carried out with the emulsion has a fungicidal activity starting from a concentration of 14 mL/cm^2^, and does not induce significant colour variations and induces at most only beneficial variations (alkalinization) in terms of pH of oil canvases performed on both priming and chalk and glue bases. Whereas samples formulated with tempera on primer showed significant colour variations (ΔE > 3) probably due to the lower permeability of the pictorial base that does not favour a perfect adhesion of the tempera during the ageing phase.

Preliminary in vitro data made it possible to move on to in situ treatment with reasonable certainty regarding the safety of the spray treatment when carried out on ancient oil paintings. Additionally, the in situ experiment carried out on the painting “Il Silenzio” did not show visual alterations. No mechanical or aesthetical visual changes were determined by treatment, canvas fibres were homogeneous and compact, and the painted surface did not show visual variations in tone and chromatic saturation after treatment. In addition, the type of treatment and the materials used did not hinder the subsequent restoration phases necessary for the painting.

Moreover, the health aspect of the product must not be forgotten, and restoration treatments must also be considered and discussed in terms of safety for restorers. As it is known from the literature, the levels of organic solvent vapours present in the workplace air during the restoration practices were found to be well above the occupational exposure limits, thus constituting a threat to the health of the worker [32]. This chemical exposure together with the presence of fungal contaminants in the bioaerosols of museum environments are recognised as a source of occupational diseases affecting the upper respiratory tract of restorers [33,34].

Therefore, it would be of great importance to identify fungicidal products that are at the same time effective, easy to use, and safe for restorers’ health. Our in vivo studies conducted on *Galleria mellonella* indicated that the emulsion based on *C. aurantium* var. *amara* Hy and *C. zeylanicum* EO was reasonably safe because, after 3 days of treatment, the in vivo experiment did not show toxicity against any of the ten larvae which, on the contrary, were all perfectly viable. Furthermore, the emulsion used in this study was composed of 99.7% *C. aurantium* var. *amara* Hy regularly marketed without any restriction for human use and 0.3% of *C. zeylanicum* (bark) EO of which it is important to control the concentration of two active ingredients (Safrole and Cinnamaldehyde), which are regulated in formulations for human use by the International Fragrance Association (IFRA) [35]. In particular, the IFRA standards indicate that EOs containing safrole should not be used at a level such that the total concentration of safrole, isosafrole, and dihydrosafrole exceeds 0.01% in consumer products [36]. The studied emulsion had a safrole content equal to 0.00018% v/v, enormously below that allowed by the IFRA recommendations. Similarly, IFRA standards recommend a cinnamaldehyde concentration between 0.014% and 1.8%, if the product is intended for human use, and do not pose any restrictions on products not intended for direct skin contact or with minimal or insignificant transfer to skin. The tested emulsion (cinnamaldehyde content equal to 0.02%) falls into the latter category because, due to the negligible skin exposure (especially reduced using personal protective equipment required by best practices), the risk of inducing sensitisation through the normal formulation and the use of such products is considered derisory [35,37].

This study fits perfectly into a world that is globally shifting its attention to green approaches in the various productivity sectors to protect human health and the environment. In conclusion, the introduction of a product of natural origin with outstanding fungicidal activity in the restoration practices could be beneficial both to preserve artistic heritage and to protect the health of the restorers.

## Figures and Tables

**Table 1:** Description of preparatory and pictorial layers.

Ancient Techniques			
Sample	Technique	Preparation	Paint Layer
T1	Tempera	Priming: white lead (zinc white) 12.5 g, litharge. (minium) 12.5 g, black of vine 0.5 g, boiled linseed oil 14 g	Egg-red tempera and respective pigments: red earth, ultramarine blue, green earth, yellow ochre
T2	Tempera	Bologna chalk and 1:9 rabbit glue	Egg-red tempera and respective pigments: red earth, ultramarine blue, green earth, yellow ochre
O1	Oil	Priming: white lead (zinc white) 12.5 g, litharge. (minium) 12.5 g, black of vine 0.5 g, boiled linseed oil 14 g	Boiled linseed oil and respective pigments: red earth, ultramarine blue, green earth, yellow ochre
O2	Oil	Bologna chalk and 1:9 rabbit glue	Boiled linseed oil and respective pigments: red earth, ultramarine blue, green earth, yellow ochre

**Table 2:** Chemical composition (%) of *C. zeylanicum* EO.

N°	Component ^1^	LRI ^2^	LRI ^3^	EO (%) ^4^
1	*α*-thujene	920	923	0.1
2	*α*-pinene	938	943	2.7
3	camphene	942	946	0.2
4	*β*-pinene	985	986	0.2
5	*α*-phellandrene	998	996	0.4
6	p-cymene	1020	1026	3.8
7	1,8-cineole	1022	1027	4.4
8	*γ*-terpinene	1050	1054	tr
9	cis-linalool oxide	1060	1058	tr
10	linalool	1089	1092	4.9
11	camphor	1130	1126	0.1
12	*α*-terpineol	1019	1021	0.1
13	4-terpinenyl acetate	1283	1286	0.1
14	*O*-anisaldheyde	1250	1242 *	0.1
15	cinnamaldheyde	1269	1275	66.0
16	eugenol	1333	1331	4.1
17	β-caryophyllene	1429	1426	5.8
18	(*E*)-cinnamyl acetate	1441	1439	5.5
19	humulene	1450	1454	0.4
20	eugenol acetate	1480	1483	0.1
21	O-metoxy cinnamaldheyde	1507	1505	0.3
22	δ-cadinene	1529	1530	0.1
23	caryophyllene oxide	1577	1583	0.3
24	benzyl benzoate	1741	1739	0.3
	SUM (%)			100.0
	Monoterpene hydrocarbons			7.4
	Oxygenated monoterpenes			9.6
	Sesquiterpene hydrocarbons			6.3
	Oxygenated sesquiterpene			0.3
	Others			76.4

^1^ the components are reported according to their elution order on apolar column ^2^ Linear Retention indices measured on apolar column; ^3^ Linear Retention indices from literature; *: Normal Alkane retention index; ^4^ Percentage mean values of *C. zeylanicum* EO components (%); tr: traces < 0.1.

**Table 3:** Fungistatic (MIC) and fungicidal (MFC) activity of hydrolates and essential oils against fungal strains.

Essential Oil	Fungal Strain	MIC	MFC
*C. zeylanicum*	*Alternaria alternata*	0.031%	0.031%
	*Aspergillus niger* ATCC 9642	0.125%	0.125%
	*Aureobasidium pullulans* ATCC 15233	0.031%	0.031%
	*Chaetomium globosum* ATCC 6205	0.008%	0.008%
	*Cladosporium cladosporioides* ATCC 16022	0.008%	0.008%
	*Penicillium citrinum*	0.125%	0.125%
*M. didyma*	*Alternaria alternata*	2%	2%
	*Aspergillus niger* ATCC 9642	4%	4%
	*Aureobasidium pullulans* ATCC 15233	2%	2%
	*Chaetomium globosum* ATCC 6205	4%	>4%
	*Cladosporium cladosporioides* ATCC 16022	4%	>4%
	*Penicillium citrinum*	4%	4%
*M. fistulosa*	*Alternaria alternata*	1%	1%
	*Aspergillus niger* ATCC 9642	1%	2%
	*Aureobasidium pullulans* ATCC 15233	4%	>4%
	*Chaetomium globosum* ATCC 6205	4%	>4%
	*Cladosporium cladosporioides* ATCC 16022	2%	2%
**Hydrolats**	**Fungal Strains**	**MIC**	**MFC**
*C. aurantium* var. *amara*	*Alternaria alternata*	1.56%	1.56%
	*Aspergillus niger* ATCC 9642	6.25%	6.25%
	*Aureobasidium pullulans* ATCC 15233	3.125%	3.125%
	*Chaetomium globosum* ATCC 6205	1.56%	1.56%
	*Cladosporium cladosporioides* ATCC 16022	1.56%	1.56%
	*Penicillium citrinum*	1.56%	1.56%
*M. citriodora*	*Alternaria alternata*	12.5%	12.5%
	*Aspergillus niger* ATCC 9642	50%	50%
	*Aureobasidium pullulans* ATCC 15233	12.5%	12.5%
	*Chaetomium globosum* ATCC 6205	12.5%	12.5%
	*Cladosporium cladosporioides* ATCC 16022	6.25%	6.25%
	*Penicillium citrinum*	≥50%	≥50%
*M. didyma*	*Alternaria alternata*	≥50%	≥50%
	*Aspergillus niger* ATCC 9642	≥50%	≥50%
	*Aureobasidium pullulans* ATCC 15233	≥50%	≥50%
	*Chaetomium globosum* ATCC 6205	≥50%	≥50%
	*Cladosporium cladosporioides* ATCC 16022	6.25%	3.125%
	*Penicillium citrinum*	≥50%	≥50%
*M. fistulosa*	*Alternaria alternata*	25%	25%
	*Aspergillus niger* ATCC 9642	≥50%	≥50%
	*Aureobasidium pullulans* ATCC 15233	25%	25%
	*Chaetomium globosum* ATCC 6205	12.5%	12.5%
	*Cladosporium cladosporioides* ATCC 16022	12.5%	12.5%
	*Penicillium citrinum*	≥50%	≥50%

**Table 4:** pH values of canvases before and after treatment.

Colour	Technique	Unaged				Aged			
		A-BT ^1^	A-AT ^2^	AV ^3^	SD ^4^	A-BT	A-AT	AV	SD
Red	T1 ^5^	6.5	5.6	−0.9 *	**0.4**	4.5	5.0	**0.5 ns**	**0.4**
	T2 ^6^	5.3	5.7	**0.3 ns**	**0.2**	4.1	4.7	**0.5 ^(+)^**	**0.3**
	O1 ^8^	5.8	5.8	**0.0 ns**	**0.1**	5.4	6.2	0.8 **	**0.1**
	O2 ^7^	5.4	5.6	**0.2 ns**	**0.2**	4.8	4.9	**0.2 ns**	**0.1**
Blue	T1	6.0	5.7	**−0.3 ns**	**0.2**	4.5	5.3	0.8 *	**0.3**
	T2	5.4	5.7	0.3 *	**0.0**	4.7	5.1	0.4 *	**0.1**
	O1	6.1	5.9	**−0.3 ns**	**0.3**	5.3	6.4	1.0 **	**0.1**
	O2	5.7	5.5	**−0.2 ns**	**0.4**	4.6	4.9	0.3 **	**0.1**
Green	T1	5.7	5.4	**−0.3 ns**	**0.2**	4.4	4.8	0.4 **	**0.1**
	T2	5.5	5.7	**0.2 ^(+)^**	**0.1**	4.8	5.0	**0.3 ^(+)^**	**0.1**
	O1	6.1	5.9	**−0.2 ns**	**0.2**	5.8	6.2	0.4 *	**0.1**
	O2	6.0	5.7	**−0.3 ns**	**0.3**	6.2	5.7	−0.5 *	**0.2**
Ocher	T1	5.6	4.9	**−0.7 ns**	**1.0**	4.7	4.6	**0.0 ns**	**0.1**
	T2	5.8	5.8	**0.0 ns**	**0.2**	4.8	5.2	0.4 *	**0.1**
	O1	6.1	6.1	**0.0 ns**	**0.2**	5.5	6.3	0.8 **	**0.2**
	O2	5.5	5.6	**0.1 ns**	**0.2**	5.8	6.0	**0.2 ^(+)^**	**0.1**

Note. ^1^ Average before treatment, ^2^ Average after treatment, ^3^ Average variation, ^4^ Standard deviation, ^5^ Tempera on priming, ^6^ Tempera on chalk and glue, ^7^ Oil on priming, ^8^ Oil on chalk and glue. **ns**: non-significant; ^(+)^: *p* < 0.10; *: *p* < 0.05; **: *p* < 0.01 (*t*-test).

**Table 5:** Mean values of colorimetric parameters.

		Unaged Canvas (Mean Values)								Aged Canvas (Mean Values)							
		Before Treatment			After Treatment					Before Treatment			After Treatment				
Colour	Sample	L*	a*	b*	L*	a*	b*	DE	SD-DE ^1^	L*	a*	b*	L*	a*	b*	DE	SD-DE
Red	T1 ^2^	34.2	18.2	14.4	35.2	19.1	15.4	**1.8**	**0.4**	47.7	9.4	4.9	40.0	13.4	10.1	**17.8**	**17.0**
	T2 ^3^	35.2	18.5	15.1	35.2	18.6	15.4	**0.7**	**0.4**	34.9	18.9	15.1	34.8	19.0	15.0	**0.4**	**0.2**
	O1 ^4^	35.0	21.1	19.0	34.3	21.0	19.1	**1.4**	**0.6**	32.8	19.6	18.3	34.2	20.1	19.0	**2.0**	**0.2**
	O2 ^5^	33.3	15.6	12.5	33.3	15.5	12.5	**0.5**	**0.2**	31.4	19.8	18.2	32.1	20.1	18.5	**0.8**	**0.2**
Blue	T1	25.1	9.7	−24.2	24.9	9.2	−23.7	**0.9**	**0.5**	40.7	5.9	−10.0	40.7	5.9	−10.0	**7.7**	**4.6**
	T2	28.3	29.6	−56.0	28.1	28.6	−54.6	**1.7**	**0.2**	30.2	21.2	−40.3	30.2	21.2	−40.3	**7.2**	**9.6**
	O1	25.8	6.5	−19.9	25.9	6.3	−18.9	**1.0**	**0.1**	27.8	8.2	−29.3	27.8	8.2	−29.3	**2.6**	**0.5**
	O2	26.9	14.2	−36.7	26.6	13.2	−35.1	**1.7**	**1.4**	34.3	21.5	−56.4	34.3	21.5	−56.4	**1.9**	**0.5**
Green	T1	36.9	−5.4	10.6	36.5	−5.3	10.7	**0.7**	**0.4**	41.3	−1.9	8.8	41.3	−1.9	8.8	**3.2**	**1.4**
	T2	35.0	−5.8	9.9	35.2	−5.7	9.8	**0.3**	**0.1**	36.6	−3.2	11.0	36.6	−3.2	11.0	**1.3**	**0.8**
	O1	32.7	−3.8	7.9	33.0	−3.6	8.0	**0.4**	**0.2**	46.5	−4.7	11.0	46.5	−4.7	11.0	**0.6**	**0.2**
	O2	33.0	−4.3	6.5	32.8	−4.2	6.6	**0.3**	**0.1**	42.4	−3.8	9.6	42.4	−3.8	9.6	**0.8**	**0.3**
Ocher	T1	59.5	14.7	45.4	59.3	14.9	46.3	**0.9**	**0.2**	58.3	13.2	42.0	58.3	13.2	42.0	**1.3**	**0.6**
	T2	61.4	18.0	49.8	60.1	20.9	50.1	**0.7**	**0.4**	61.2	19.2	53.6	61.2	19.2	53.6	**2.4**	**0.5**
	O1	38.2	9.5	5.0	38.1	9.5	5.6	**0.9**	**0.5**	57.9	12.9	43.2	57.9	12.9	43.1	**1.8**	**0.4**
	O2	59.3	18.8	48.2	59.8	19.2	49.1	**0.7**	**0.3**	58.2	17.7	47.4	58.2	17.7	47.4	**1.6**	**0.8**

Note. ^1^ Standard deviation of DE, ^2^ Tempera on priming, ^3^ Tempera on chalk and glue, ^4^ Oil on priming, ^5^ Oil on chalk and glue.
